# ACx-projecting cholinergic neurons in the NB influence the BLA ensembles to modulate the discrimination of auditory fear memory

**DOI:** 10.1038/s41398-023-02384-8

**Published:** 2023-03-06

**Authors:** Yan Yan, Da Song, Yue Jin, Yujun Deng, Chunjian Wang, Tao Huang, Yuanhong Tang, Yu Yang, Yun Zhang, Zhe Wang, Zhifang Dong, Yuetian Wang, Juan Zhao, Junjun Ni, Hui Li, Jun Zhang, Yiran Lang, Yili Wu, Hong Qing, Zhenzhen Quan

**Affiliations:** 1 Key Laboratory of Molecular Medicine and Biotherapy, School of Life Science, Beijing Institute of Technology, Beijing, 100081 China; 2 Shandong Key Laboratory of Behavioral Medicine, Shandong Collaborative Innovation Center for Diagnosis, Treatment & Behavioral Interventions of Mental Disorders, School of Mental Health, Jining Medical University, Jining, 272013 China; 3 Advanced Innovation Center for Human Brain Protection, Capital Medical University; The National Clinical Research Center for Geriatric Disease, Xuanwu Hospital, Capital Medical University, Beijing, 100069 China; 4 Ministry of Education Key Laboratory of Child Development and Disorders, National Research Center for Child Health and Disorders, Chongqing Key Laboratory of Translational Medical Research in Cognitive Development and Learning and Memory Disorders, Children’s Hospital of Chongqing Medical University, Chongqing, 400014, China; 5 Beijing Advanced Innovation Center for Intelligent Robots and Systems, Beijing Institute of Technology, Beijing, 100081 China; 6 Key Laboratory of Alzheimer’s Disease of Zhejiang Province, School of Mental Health, Institute of Aging, Wenzhou Medical University, Wenzhou, Zhejiang 325000 China

**Keywords:** Learning and memory, Physiology

## Abstract

Animals need discriminating auditory fear memory (DAFM) to survive, but the related neural circuits of DAFM remain largely unknown. Our study shows that DAFM depends on acetylcholine (ACh) signal in the auditory cortex (ACx), which is projected from the nucleus basalis (NB). At the encoding stage, optogenetic inhibition of cholinergic projections of NB-ACx obfuscates distinct tone-responsive neurons of ACx recognizing from fear-paired tone to fear-unpaired tone signals, while simultaneously regulating the neuronal activity and reactivation of basal lateral amygdala (BLA) engram cells at the retrieval stage. This NB^ACh^-ACx-BLA neural circuit for the modulation of DAFM is especially dependent on the nicotinic ACh receptor (nAChR). A nAChR antagonist reduces DAFM and diminishes the increased magnitude of ACx tone-responsive neuronal activity during the encoding stage. Our data suggest a critical role of NB^ACh^-ACx-BLA neural circuit in DAFM: manipulation of the NB cholinergic projection to the ACx via nAChR during the encoding stage affects the activation of ACx tone-responsive neuron clusters and the BLA engram cells during the retrieval stage, thus modulating the DAFM.

## Introduction

Survival in nature requires animals to develop rapid fear responses to cope with threatening environments. To do so, animals must discriminate between different sensory stimuli (images, smells, sounds) and associate only relevant ones with threatening signals [1–3]. In Pavlovian experiments of auditory fear memory (AFM), animals learn to fear a neutral sound stimulus and display conditioned fear responses when the neutral sound stimulus is coupled with an aversive or threatening stimulus. However, they do not respond to or fear other irrelevant auditory stimuli [4–6]. At the neural circuit level, how animals selectively discriminate auditory stimuli that lead to fear responses is poorly understood.

Existing research shows that cholinergic neurons in the basal forebrain (BF) act as an extra-thalamic relay system for sensory information and regulate fear memory [7, 8]. The amygdala, which is critical for fear memory, receives cholinergic signals from the BF. Both pyramidal neurons and interneurons in the BLA receive rich cholinergic inputs, which originate mainly from the nucleus basalis (NB) and the horizontal branch of Broca’s diagonal band (DB) [5, 9–11]. Optogenetic stimulation of cholinergic signaling from the NB/DB can lead to BLA modulation by inducing the firing of putative BLA pyramidal neurons, suppressing or activating the neuronal activity, and inducing long-term potentiation (LTP) in cortical-BLA circuits for the control of fear memory extinction [12]. Furthermore, cholecystokinin (CCK) neurons that are innervated by BF cholinergic neurons mediate cholinergic-induced di-synaptic inhibition of BLA pyramidal neurons [13]. Cholinergic axons originating from the caudal BF can modulate auditory cortical tuning via targeting both excitatory and inhibitory auditory cortical cell types, leading fast movement-related activity [14–16]. However, whether cholinergic signals in the NB would affect the ACx-BLA circuit to modulate of the DAFM remain unclear.

In this study, we observed that ACh signaling from the NB to ACx is responsive to and required for DAFM, which is dependent on nicotinic ACh receptors (nAChRs). During the encoding stage, an nAChR antagonist reduced the discriminative ability of AFM in mice. During the retrieval stage, optogenetic inhibition of cholinergic projections of NB-ACx obfuscates distinct tone-responsive neurons of ACx in recognizing fear-paired tone from fear-unpaired tone signals, while simultaneously changing the neuronal activity and reactivation of BLA engram cells. This suggests the critical role of NB^ACh^-ACx-BLA circuit for the modulation of DAFM.

## Results

### The acetylcholine signal in the ACx is responsive to the DAFM

**Fig. 1:**
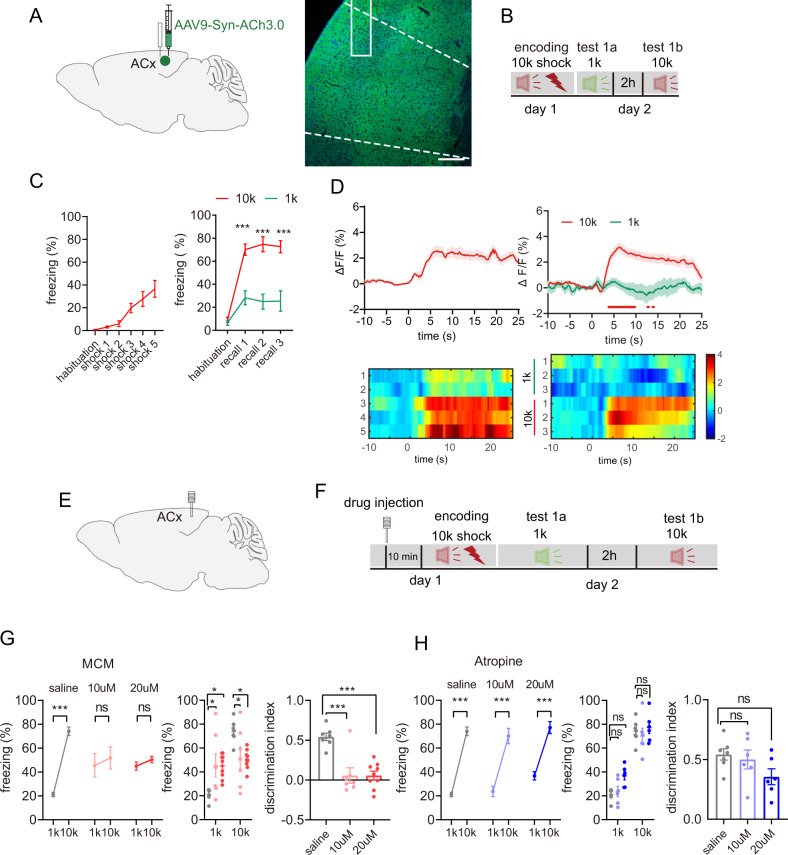
The ACh signaling in the ACx participates in the DAFM via nAChR. **A** Schematic of virus injection of AAV9-Syn-ACh3.0 and representative confocal images of ACh3.0 expression in the ACx (blue: DAPI; green: ACh3.0) Scale bar: 200 μm. **B** Behavioral design. **C** The behavioral performances on day 1 and day 2 (right, F (3, 48) = 7.588, *P* < 0.001, *n* = 7). **D** The mean and SEM of the fluorescence responses of ACh3.0 in the ACx of mice at day 1 (left) and day 2 (right) and their corresponding heatmaps. The red line indicated bins between the 10k and 1k trials with two-tailed unpaired t-test significance (*P* < 0.05). **E** Schematic of bilateral cannulas injection. **F** Behavioral design. **G** Percentage of the freezing time (left, middle) and the discrimination index (right) in mice under the treatment of nAChR blocker mecamylamine (MCM) in the ACx at the encoding stage (saline, *n* = 7; 10 µM, *n* = 6; 20 µM, *n* = 6; freezing: F (2, 40) = 11.24, *P* < 0.001; discrimination index: F (2, 20) = 16.65, *P* < 0.0001). **H** Percentage of the freezing time (left, middle) and the discrimination index (right) in mice under treatment of mAChR blocker Atropine in the ACx at the encoding stage (saline, *n* = 7; 10 µM, *n* = 7; 20 µM, *n* = 9; freezing: F (2, 40) = 11.24, *P* < 0.001; discrimination index: F (2, 16) = 2.337, *P* = 0.13). Statistical tests: (**C**, **G**, and **H**) freezing: two-way ANOVA with Bonferroni’s multiple comparisons tests. **G**, **H** discrimination index: one-way ANOVA with Bonferroni’s multiple comparisons tests. ns not significant; *P < 0.05, **P < 0.01, ***P < 0.001. Data are presented as mean ± SEM.

To find out whether the discriminative ability of auditory fear memory can be observed in mice, we first performed an optimized behavioral test, which was previously reported [4]. Mice were delivered foot shocks (5 times) paired with a tone of a specific frequency (10 kHz) during the encoding stage on day 1. During the retrieval stage on day 2, mice were sequentially exposed to three times of a shock-unpaired, frequency 1 kHz tone (test 1a). They were then exposed to three times of a shock-paired, 10 kHz tone (test 1b) at 2-h intervals (Fig. 1B). On day 1, mice showed gradually increasing freezing time in response to the repetition of the 10 kHz tone, which was paired with foot shocks. On day 2, mice exhibited a more significant freezing response to the shock-paired 10 kHz tone than to the shock-unpaired 1 kHz tone (Fig. 1C), suggesting that mice can discriminate threatening shock-paired signals from neutral shock-unpaired signals. To exclude the possibility that the responses of mice were due to the influence of different frequency tones in DAFM, mice were delivered footshocks with a 1 kHz tone at the encoding stage (day 1) and then delivered both a 10 kHz tone and a 1 kHz tone at 2-hour intervals at the retrieval stage (day 2) (Fig. S1A). It was observed that the mice showed an increased freezing time on day 1 to the 1 kHz tone. On day 2, during the retrieval stage, the mice displayed a longer freezing time at the 1 kHz retrieval stage than at the 10 kHz retrieval stage (Fig. S1B). The above data indicate that the discriminative ability of mice is not based on the frequency of tones but on fear-paired cues.

The ACx is a major site for sensory processing and plays an important role in DAFM [14]. To find out whether or not the ACh signal in the ACx is involved in DAFM, we firstly recorded the ACh signal in the ACx by injecting AAV9-Syn-ACh3.0 virus as an ACh sensor in the ACx (Fig. 1A). The ACh signal gradually increased during the encoding stage when mice were delivered with foot shocks coupled with a 10 kHz tone on day 1 (Fig. 1D left). On day 2, the ACh signal was significantly lower under the 1 kHz tone retrieval than under the 10 kHz tone retrieval (Fig. 1D right), indicating that ACh signaling does participate in DAFM.

To further determine the role of ACh signal in DAFM, we inhibited the ACh signal by infusing ACh receptor blockers in the ACx at the encoding stage (Fig. 1E, F). Administration of the nicotinic ACh receptor (nAChR) blocker mecamylamine (MCM) significantly decreased the discriminative index (Fig.1G and Fig. S1D). When the 10 kHz tone was paired with footshocks, the treatment of MCM dramatically increased the freezing time of mice under the 1 kHz tone retrieval and decreased the freezing time of mice under the 10 kHz tone retrieval (when compared to the control group) (Fig. 1G). On the other hand, the treatment of atropine (AP), an antagonist of muscarinic ACh receptor (mAChR), did not affect the discrimination (Fig. 1H). In addition, when the 1 kHz tone was paired with footshocks, we also found that the treatment of MCM dramatically increased the freezing time under the 10 kHz retrieval, but decreased under the 1 kHz retrieval, thus leading to a reduced discriminative index (Fig. S1C, D). These results show that the nAChR of the ACx is specifically responsible for the discriminative ability of AFM in mice.

### NB cholinergic projection to the ACx is required for DAFM

The NB has a strong cholinergic projection to the ACx [14]. We propose that the ACh signal of the ACx might originate from the NB cholinergic neurons and influence DAFM. To test this, we first tested whether the observed ACh signal of the ACx originates from NB cholinergic neurons and found that activity of the ACx neurons (Fig. S2B) and the acetylcholine level in the ACx was upregulated via activating cholinergic neurons in the NB (Fig. S2C).

**Fig. 2:**
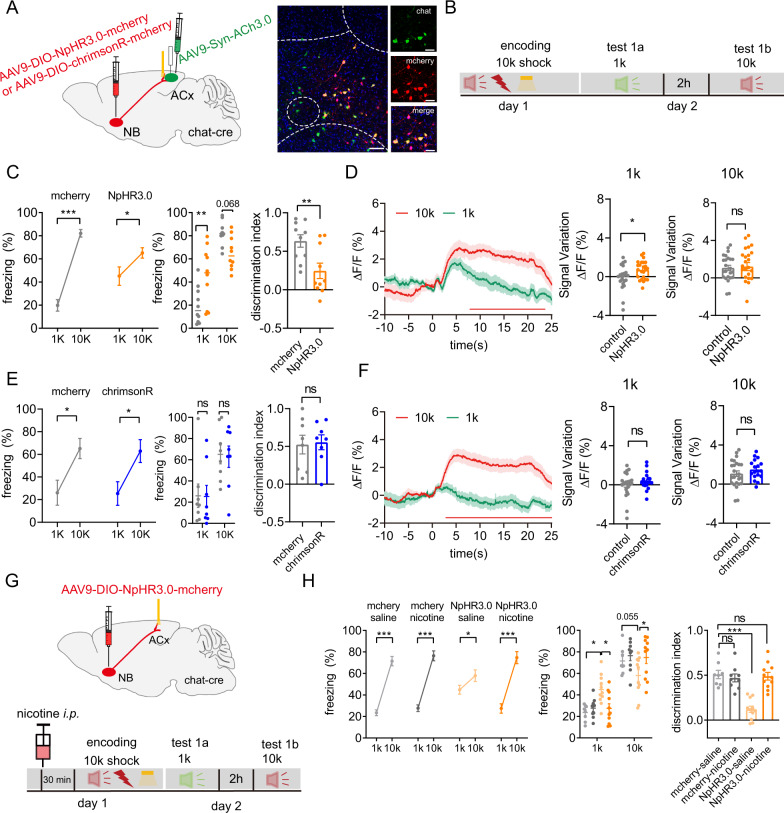
Optogenetic manipulation of ACh signal in the ACx at the encoding stage regulates the DAFM. **A** Left, schematic of virus injection. In ChAT-cre mice, AAV9-DIO-NpHR3.0 or AAV9-DIO-ChrimsonR-mcherry was infused into the NB; AAV9-Syn-ACh3.0 was infused into the ACx. Right: Confocal images of coronal sections showing Cre^+^ neurons (mcherry^+^) in the NB of ChAT-cre mice were co-labeled with cholinergic neurons (green) Scale bar: 200 μm; 20 μm. **B** Behavioral design. **C**, **E** Percentage of the freezing time (left, middle) and discrimination index (right) in mice under inhibition (**C**) or activation (**E**) of ACx-projecting cholinergic neurons of the NB at the encoding stage (**C**, NpHR3.0, *n* = 10, mCherry, *n* = 10; F (1, 36) = 13.96, *P* < 0.001; **E** ChrimsonR, *n* = 8; mCherry, *n* = 8; F (1, 28) = 0.0059, *P* = 0.94). **D**, **F** Left: The mean and SEM of the fluorescence responses of ACh3.0 in the ACx of mice at day 2 receiving inhibition (**D**) or activation (**F**) of the ACx-projecting cholinergic neurons of the NB at the encoding stage. Red line indicated bins between the 10k and 1k trials with two-tailed unpaired t-test significance (*P* < 0.05). Middle and Right: quantification of the average amplitude of increased signal against baseline between control (Fig. 1D, right) and NpHR3.0 (Fig. 2D, left) or ChrimsonR (Fig. 2F, left) group during the 1 kHz tone retrieval (middle) or 10 kHz tone retrieval (right). **G** Schematic of virus injection of AAV9-DIO-NpHR3.0-mcherry in the NB and behavioral design. **H** Percentage of the freezing time (left, middle) and discrimination index (right) in mice under either or both nicotine intraperitoneal injection and inhibition of ACx-projecting cholinergic neurons of NB at the encoding stage (mCherry-saline, *n* = 8; mCherry-nicotine, *n* = 9; NpHR3.0-saline, *n* = 11; NpHR3.0-nicotine, *n* = 11; freezing: F (3, 70) = 7.862, *P* < 0.001; discrimination index: F (3, 35) = 21.02, *P* < 0.001). Statistical tests: **C**, **E**, and **H**, freezing: two-way ANOVA with Bonferroni’s multiple comparisons tests. **C**, **E** discrimination index: unpaired two-tailed *t*-test. **H** discrimination index: one-way ANOVA with Bonferroni’s multiple comparisons tests. **D**, **F** signal variation: unpaired two-tailed Mann–Whitney-*U*-test. ns not significant; *P < 0.05, **P < 0.01, ***P < 0.001. Data are presented as mean ± SEM.

To assess the contribution of ACh signaling in the ACx to the DAFM, we optogenetically inhibited or activated NB cholinergic neural terminals projecting to ACx in ChAT-cre mice during the encoding stage of the associative learning paradigm by implanting optic-fiber targeted to the ACx (Fig. 2A, B). Inhibition of NB cholinergic terminals in the ACx during the encoding stage resulted in a significantly reduced discrimination index in the DIO-NpHR3.0 group mice which was reflected by the increased freezing time in the fear-unpaired 1 kHz tone retrieval stage and slightly reduced freezing time in the fear-paired 10 kHz tone retrieval stage, as compared to the control group (Fig. 2C and Fig. S2D). The decreased discrimination index indicates that the discriminative ability of mice to distinguish shock-paired tones from shock-unpaired tones was reduced by the inhibition of the NB cholinergic projection to the ACx. Correspondingly, the ACh signal in the ACx of the DIO-NpHR3.0 group mice increased under the 1 kHz tone retrieval stage but did not change much under the 10 kHz tone retrieval stage when compared to the control group (Fig. 2D, related to Fig. 1D right). These results suggest that inhibition of the NB-ACx cholinergic projection at the encoding stage reduced the discriminative ability of AFM in mice. However, with activation of NB cholinergic terminals in the ACx during the encoding stage, the ChrimsonR group mice exhibited a similar discrimination index (Fig. 2E) and unchanged level of ACh signal (Fig. 2F, related to Fig. 1D right) when compared to the control group. This could be because the discriminative ability for auditory fear memory of mice could not be further enhanced by activating the NB-ACx projection. However, given that nicotine acts as an agonist of nAChR, treatment of nicotine coupled with optogenetic inhibition of the NB cholinergic terminals in the ACx (DIO-NpHR3.0-nicotine group) produced a restored discrimination index compared to the DIO-NpHR3.0 group. The nicotine-only group exhibited unchanged fear discrimination (Fig. 2G, H and Fig. S2E). These data indicate that nicotine can restore the decreased DAFM caused by optogenetic inhibition of the NB-ACx projection at the encoding stage.

We next examined whether inhibition of the NB-ACx projection at the retrieval stage would affect DAFM. We found that the discrimination index was not affected in the DIO-NpHR3.0 group of mice compared to the control group of mice (Fig. S2F–H), strongly indicating that DAFM is affected by inhibition of the NB-ACx projection at the encoding stage, rather than at the retrieval stage. Our data further show that the ACx-projecting cholinergic signal in the NB at the encoding stage is required for DAFM.

### Tone-responsive ACx neurons regulate the BLA engram activity

**Fig. 3:**
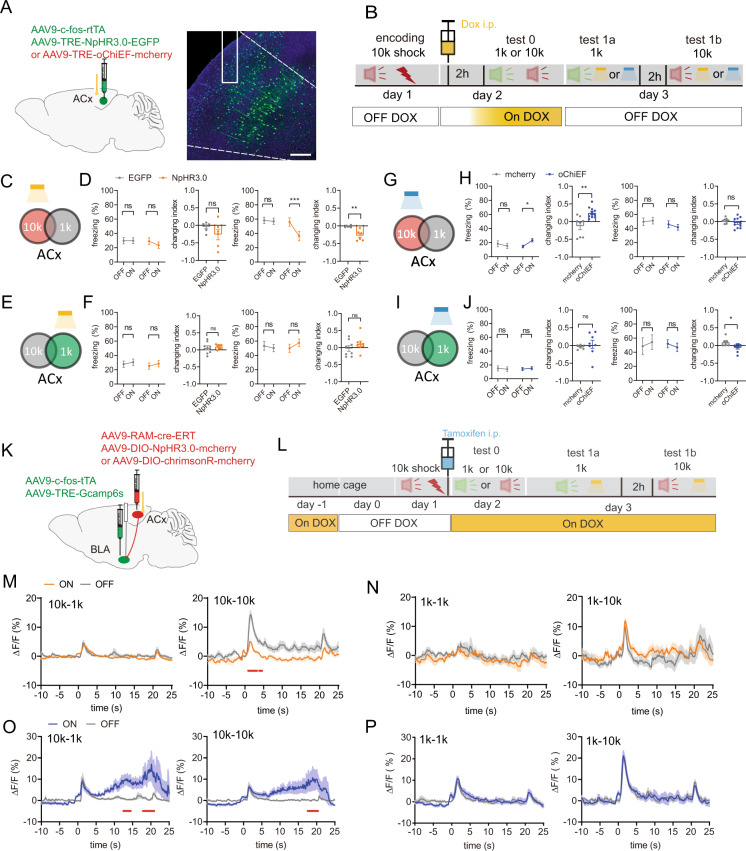
Optogenetic manipulation of tone-responsive neurons in ACx modulates BLA engram cells to regulate DAFM. **A** Schematic of virus injection. AAV9-c-fos-rtTA and AAV9-TRE-NpHR3.0-EGFP or AAV9-TRE-oChiEF-mcherry were infused into the ACx and representative confocal images of NpHR3.0 expression in the ACx. Scale bar: 200 μm. **B** Behavioral design. **C**, **E**, **G**, and **I** Schematic of optogenetic stimulation. **D**, **F**, **H**, **J** The percentage of the freezing time (1, 3) and the changing index (2, 4) in mice during 1 kHz retrieval stage (1, 2) or 10 kHz retrieval stage (3, 4) at day 3 when optogenetic inhibition (**D**, **F**) and activation (**H**, **J**) of the 10 kHz-tone responsive ACx neurons (**D**, **H**) and 1 kHz-tone responsive ACx neurons (**F**, **J**) (**D** NpHR3.0, *n* = 6, EGFP, *n* = 6; **F** NpHR3.0, *n* = 8, EGFP, *n* = 8; **H** oChiEF, *n* = 11, mCherry, *n* = 8; **J** oChiEF, *n* = 8, mCherry, *n* = 6). (**K**), Schematic of virus injection. AAV9-c-fos-tTA and AAV9-TRE-Gcamp6s were infused into the BLA, and AAV9-RAM-cre-ERT, AAV9-DIO-NpHR3.0-mcherry or AAV9- DIO-ChrimsonR-mCherry were infused into the ACx. Optical fiber was implanted in the ACx. Gcamp6s was injected to record neural activity of BLA engram cells by optical fiber. **L** Behavioral design. **M**–**P** The mean and SEM of the corresponding Ca^2+^ signal of engram cells in the BLA during 1 kHz retrieval stage (**M**–**P**, left) or 10 kHz retrieval stage (**M**–**P** right) at day 3 when inhibiting or activation of the 10 kHz-tone-responsive ACx neurons (**M**, **O**) or 1 kHz-tone-responsive ACx neurons (**N**, **P**). **l** *n* = 6; m, *n* = 7). The under red line indicated bins between the light-on and light-off trials, with two-tailed unpaired t-test significance (*P* < 0.05). Statistical tests: **D**, **F**, **H**, and **J**, freezing: two-way ANOVA with Bonferroni’s multiple comparisons tests; **D**, **F**, **H**, and **J**, changing index: unpaired two-tailed *t*-test. ns not significant; *P < 0.05, **P < 0.01, ***P < 0.001. Data are presented as mean ± SEM.

We understood that inhibition of the NB-ACx projection at the encoding stage influences the discriminative ability of mice during the retrieval stage, but how the ACx neurons would change during the retrieval stage was yet unknown. In neutral conditions without fear memory, ACx can modulate auditory tone responses and distinguish different frequencies of pure-tone auditory stimuli via activating different neuronal ensembles in ACx [17]. However, during the retrieval stage of fear memory discrimination, whether different neuronal ensembles regulate the fear-paired and fear-unpaired tone information was still unclear. Therefore, we first applied the rtTA tracking system to label tone-responsive ACx neurons. The rtTA system uses doxycycline (Dox) to induce c-fos gene expression in ACx ensembles, which are activated during tasks (Fig. 3SA and B). We then injected AAV9-c-fos-rtTA and AAV9-TRE-NpHR3.0-EGFP or AAV-TRE-oChiEF-mcherry viruses to label tone-responsive neurons in the ACx to manipulate their neural activity optogenetically (Fig. 3A, B). After mice were given footshocks coupled with a 10 kHz tone at the encoding stage (day 1), mice were given either a shock-paired 10 kHz or shock-unpaired 1 kHz tone on day 2 (test 1), during which Dox was administrated to label 10 kHz- or 1 kHz-responsive neurons under the rtTA system. The mice in the TRE-NpHR3.0 and oChiEF groups did not show any differences in freezing times compared to the EGFP control group when either 1 kHz or 10 kHz tone was delivered on day 1 or day 2 (Fig. S3G–J). When shock-paired 10 kHz-responsive ACx neurons were inhibited at the retrieval stage (day3), the freezing time of the TRE-NpHR3.0 group mice was dramatically reduced under the 10 kHz tone retrieval stage, which resulted in a reduced changing index in comparison to the control group (Fig. 3D, right 1, 2). The changing index was not affected under the 1 kHz tone retrieval stage (Fig. 3D, left 1, 2). However, inhibition of shock-unpaired 1kHz-responsive neurons in the ACx in the TRE-NpHR3.0 group mice did not affect the changing index at both the 1 kHz tone- and 10 kHz tone-retrieval stages (Fig. 3F). Inhibition of shock-unpaired ACx neuron clusters did not affect the freezing time following a shock-paired 10 kHz tone. In contrast, inhibition of the shock-paired ACx neuron clusters decreased the freezing time following a shock-paired 10 kHz tone. This discrepancy suggests that inhibition of shock-unpaired neuron clusters did not interfere with the fear memory evoked by shock-paired ACx neuron clusters.

When 10 kHz-responsive neurons in the ACx were activated, the oChIEF group mice exhibited increased freezing time under the 1 kHz tone retrieval stage, leading to an increased changing index compared to the control group, which was unchanged under the 10 kHz tone retrieval stage (Fig. 3H). It is considered that shock-paired 10 kHz encoded freezing memory has reached a threshold; further activation of 10 kHz-repsonsive neurons in the ACx could not further increase the fear memory by 10 kHz recall, but increase fear memory by 1 kHz recall. Meanwhile, activation of 1 kHz-responsive neurons in the ACx at day 3 did not influence the behaviors of mice in either the 1 kHz or the10kKz tone retrieval stages (Fig. 3J). These results confirm that the ACx performs its discriminating role through different neuron clusters during the retrieval stage of DAFM. It is well known that BLA engram cells are involved in fear memory recall [18–20], but it was still unclear whether different clusters of ACx neurons in the retrieval stage could regulate BLA engram cells and participate in DAFM. To demonstrate the inhibition effect of ACx tone-responsive neurons on neural activity in BLA engram cells, we labeled activated BLA neurons (BLA engram cells) at the encoding stage and different clusters of ACx neurons at the retrieval stage. We injected AAV9-RAM-cre-ERT and AAV9-DIO-NpHR3.0-mcherry to inhibit the tone-responsive neurons in the ACx, and AAV9-c-fos-tTA and AAV9-TRE-GCamp6s viruses to record neural activity of BLA engram cells (Fig. 3K, L). Here we used the cre-ERT system and the c-fos-tTA system conjunctively. The c-fos-tTA can selectively label the activated neurons in the BLA during the encoding stage without Dox (Fig. S3E, F), and cre-ERT can label the activated neurons in the ACx at the retrieval stage by the addition of Tamoxifen. The labeling efficiency was also verified (Fig. S3C, D). We observed that when 10 kHz-responsive neurons in the ACx were inhibited at the retrieval stage (day 3), neural activity of BLA engram cells decreased during the 10 kHz tone retrieval stage but was unaffected during the 1 kHz tone retrieval stage (Fig. 3M).

On the other hand, inhibition of 1kHz-responsive neurons in the ACx did not change neural activity of BLA engram cells during either the 10 kHz or the 1 kHz tone retrieval stages (Fig. 3N). In addition, we recorded neural activity of BLA engram cells when we optogenetically activated neurons at the ACx retrieval stage by injecting AAV9-RAM-cre-ERT and AAV9-DIO-chrimsonR-mcherry viruses (Fig. 3K). With the activation of 10 kHz-responsive neurons in the ACx on day 3, neural activity of BLA engram cells under both the 1 kHz and 10 kHz tone retrieval stages was significantly increased (Fig. 3O). Activation of 1kHz-responsive neurons in the ACx on day 3 did not affect the neural activity of BLA engram cells in mice during the 1 kHz or 10 kHz tone retrieval stages (Fig. 3P).

We also specifically stimulated BLA-projecting ACx engram neurons by injecting BLA with a retro-c-fos-rtTA virus and the ACx with AAV9-TRE-NpHR3.0/ chrimsonR -mcherry viruses. Injections resulted in similar results as shown in Fig. S4 (related to Fig. 3A–J). These results demonstrate that tone-responsive ACx neurons can drive neural activity of BLA engram cells and are responsible for distinguishing shock-paired auditory signals from shock-unpaired auditory signals during the retrieval stage of the DAFM.

### The ACx ensembles responsive to the DAFM is controlled by the ACh signaling in the NB

To investigate how nAChR in the ACx affects tone-responsive neurons at the encoding stage, we further recorded the neural activity of the tone-responsive ACx neurons before and after footshocks during the encoding stage with the injection of nAChR antagonist. The ACx ensembles were initially labeled by rtTA system, and their neural activity was recorded by expressing Gcamp6s (Fig. S5A). For 10 kHz tone, Dox was injected 2 h before 10 kHz delivery on day 1, the nAChR blocker MCM was administrated 2 h before the behavioral test, and footshocks paired with 10 kHz tone were delivered at day 2. Then the Ca^2+^ signal was recorded under 10 kHz delivery retrieval. It was observed that the Ca^2+^ amplitude was significantly increased in 10 kHz retrieval after footshocks compared to that before footshocks (saline groups), while MCM treatment dramatically reduced the Ca^2+^ amplitude in 10 kHz retrieval (post-shock-10 k-MCM group) compared with that before footshocks (pre-shock-10 k-saline group), and showed similar amplitude pattern as that in 10 kHz tone when MCM was administrated before footshocks (pre-shock-10 k-MCM group) (Fig. S5C). This phenomenon was also observed when 1 kHz was paired with foot-shocks (Fig. S5D). The data demonstrate that the neural activity of ACx ensembles that are responsive to the DAFM is controlled by the ACh signaling.

**Fig. 4:**
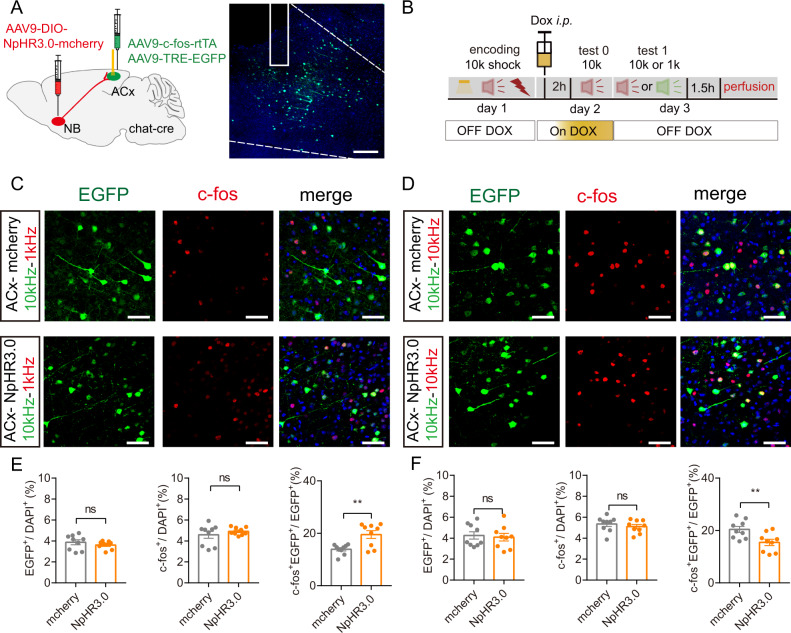
Cholinergic signaling influences the ACx neural activity to modulate tone-responsive neurons during the encoding stage. **A** Left: schematic of virus injection. In ChAT-cre mice, AAV9-DIO-NpHR3.0-mCherry was infused into the NB. The viruses AAV9-c-fos-rtTA and AAV9-TRE-EGFP were infused into the ACx. Right: representative confocal images of in the ACx. **B** Behavioral design. **C**–**F** Representative confocal images (**C** and **D**) and the statistical numbers (**E** and **F**) of EGFP^+^, c-fos^+,^ and EGFP^+^c-fos^+^ immunofluorescent cells in the ACx during 1 kHz retrieval stage (**C**, **E**) or 10 kHz retrieval stage (**D**, **F**) in mice under inhibition of ACx-projecting cholinergic neurons of NB at the encoding stage. (Blue, DAPI; green, the activated ACx cells at test 1 in day 2 that were marked by EGFP; red: the activated ACx neurons marked by c-fos in mice that are given 1 kHz tone (**C**) or 10 kHz tone (**D**) at day 3. Scale bar: 50 μm. In **E** and **F**, the NpHR3.0 and mCherry groups displayed similar percentages of EGFP^+^ and c-fos^+^ cells among DAPI^+^ cells (*n* = 9 images from three mice). In **E**, the NpHR3.0 group displayed a higher percentage of EGFP^+^c-fos^+^/EGFP^+^ cells than mCherry group during the 1 kHz retrieval test (*n* = 9 images from three mice). In **F**, the NpHR3.0 group displayed a lower percentage of EGFP^+^c-fos^+^/EGFP^+^ cells than mCherry group during the 10 kHz retrieval test (*n* = 9 images from three mice). Statistical tests: **E** and **F**, unpaired two-tailed *t*-test. ns not significant; *P < 0.05, **P < 0.01, ***P < 0.001. Data are presented as mean ± SEM.

Having determined that the NB cholinergic projection to the ACx plays a pivotal role in DAFM, we further investigated whether the optogenetic regulation of cholinergic input to the ACx at the encoding stage could influence the ACx ensembles at the retrieval stage. To find out whether NB cholinergic signaling is involved in DAFM (by influencing ACx neuronal clusters that respond to different tone signals during the retrieval stage), we applied the rtTA tracing system. The rtTA system records the ACx ensembles under inhibition of the NB-ACx projection by injecting AAV9-c-fos-rtTA and AAV-TRE-EGFP in the ACx and AAV9-DIO-NpHR3.0-mcherry in the NB of the ChAT-cre mice (Fig. 4A). With optogenetic inhibition of NB cholinergic terminals in the ACx at the encoding stage, the overlapped 10 kHz-1 kHz tone-responsive ACx neurons (EGFP^+^ c-fos^+^/EGFP^+^) of DIO-NpHR3.0 group of mice increased (Fig. 4C, E). Still, the overlapped 10 kHz-10 kHz tone-responsive ACx neurons decreased compared to the mCherry (control) group at the retrieval stage (Fig. 4D, F). This data demonstrates that the inhibition of the NB-ACx cholinergic signaling during the encoding stage affects the activation of tone-responsive ACx neurons, resulting in the confounding of distinct ACx neuronal clusters in response to different tones.

### The NB cholinergic projection to the ACx influences the BLA engram activity

**Fig. 5:**
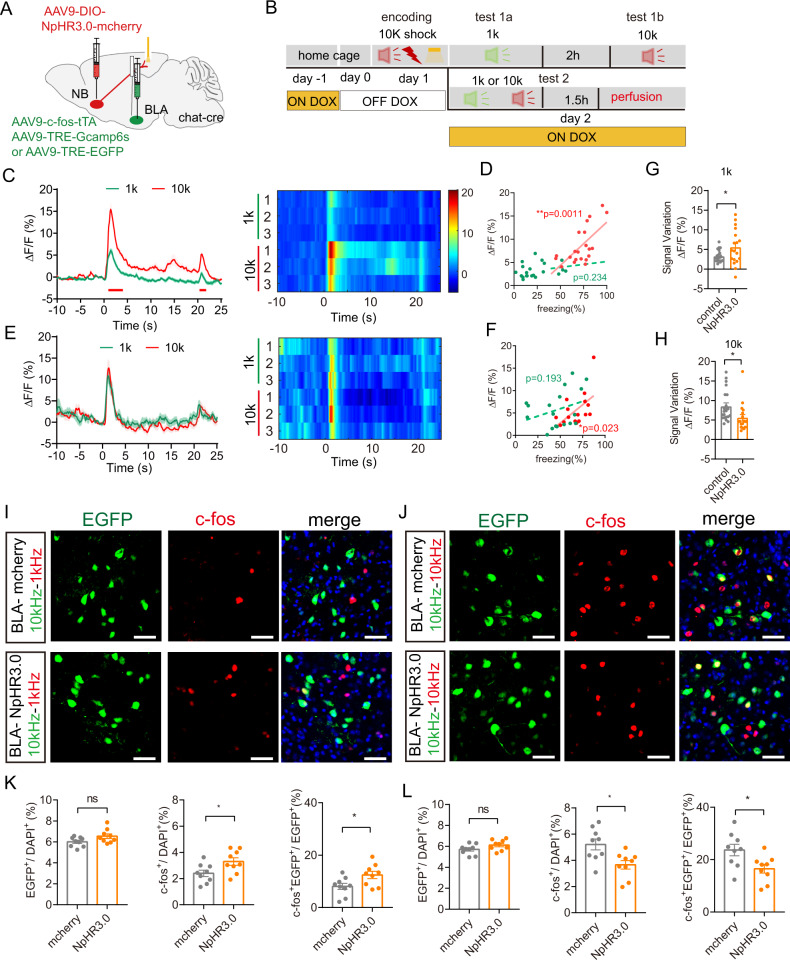
Stimulation of cholinergic inputs to ACx at the encoding stage influences the reactivation of BLA engram cells. **A** Schematic of virus injection. In ChAT-cre mice, AAV9-DIO-NpHR3.0-mCherry was infused into the NB. AAV9-c-fos-tTA and AAV9-TRE-Gcamp6s or AAV9-TRE-EGFP were infused into the BLA. **B** Behavioral design. **C**, **E** The mean and SEM of the corresponding Ca^2+^ signal of BLA engram cells in mice without (**C**) or with (**E**) inhibition of ACx-projecting cholinergic neurons of the NB at the encoding stage and their corresponding heatmaps. The under red line indicated bins between 10 kHz or 1 kHz tone retrieval, with two-tailed unpaired *t*-test significance (*P* < 0.05). **D**, **F** the correlation between freezing and the amplitudes of Ca^2+^ signal without (related to **C**) or with (related to **E**) inhibition of ACx-projecting cholinergic neurons of the NB. **G**, **H** Quantification of the averaged amplitude of increased signal against baseline between non-inhibition trials (related to **C**) and inhibition trials (related to **E**) during the 1 kHz tone retrieval (**F**) or 10 kHz tone retrieval (**H**). **I**–**L** Representative confocal images (**I** and **J**) and the statistical numbers (**K** and **L**) of the reactivated BLA engram cells during 1 kHz retrieval stage (**I** and **K**) or 10 kHz retrieval stage (**J** and **L**) in mice under inhibition of ACx-projecting cholinergic neurons of NB at the encoding stage. (Blue, DAPI; green, the activated BLA engram cells at day 1 that were marked by EGFP; red: the activated BLA neurons marked by c-fos in mice that were given 1 kHz tone (**I**) or 10 kHz tone (**J**) at day 2. Scale bar: 50 μm. In the middle graph of **K**, the NpHR3.0 group displayed higher percentages of c-fos^+^/DAPI^+^ and EGFP^+^c-fos^+^/EGFP^+^ cells than mCherry group during the 1 kHz retrieval test (*n* = 9 images from three mice). In middle graph of **L**, the NpHR3.0 group displayed lower percentages of c-fos^+^/DAPI^+^ and EGFP^+^c-fos^+^/EGFP^+^ cells than mCherry group during the 10 kHz retrieval test (*n* = 9 images from three mice). Statistical tests: **D**, **F**, correlation: two-tailed Pearson (R) correlation. **G**, **H** signal variation: unpaired two-tailed Mann–Whitney-U-test. **K**, **L** unpaired two-tailed *t*-test. ns not significant; *P < 0.05, **P < 0.01, ***P < 0.001. Data are presented as mean ± SEM.

We found that optogenetic inhibition of NB-ACx projection during the encoding stage causes obfuscation between the two activated clusters of ACx neurons during the retrieval stage. The ACx neuronal ensembles can also regulate BLA engram cells. To further determine whether inhibition of the NB-ACx projection would affect neural activity of BLA engram cells, we injected AAV9-DIO-NpHR3.0-mcherry in the NB to inhibit the NB-ACx projection, AAV-c-fos-tTA and AAV9-TRE-Gcamp6s or AAV9-TRE-EGFP to record neural activity of BLA engram cells in ChAT-cre mice (Fig. 5A). We observed that the BLA neural activity changes consistently with the behavioral performance of DAFM under normal conditions. The BLA engram cells exhibited higher evoked activity under a 10 kHz tone retrieval than that under a 1 kHz tone retrieval (Fig. 5C, D). Inhibition of the NB cholinergic terminals in the ACx decreased neural activity of BLA engrams in mice under the 10 kHz tone retrieval but increased that under the 1 kHz tone retrieval (Fig. 5E–H). Further examining the numbers of reactivated BLA engram cells, we also found that, under inhibition of the NB-ACx projection at the encoding stage, the reactivated BLA engram cells (EGFP^+^c-fos^+^/EGFP^+^) increased under the 1 kHz tone retrieval, but decreased under the 10 kHz tone retrieval in mice (Fig. 5I–L). This data indicates that inhibition of the NB cholinergic projection to ACx at the encoding stage may reduce neural activity and reactivation of BLA engram cells, leading to a reduced discriminative ability of AFM.

To determine whether or not NB could directly project to BLA and influence DAFM, we injected AAV9-DIO-NpHR3.0-mcherry virus in the NB and input opto-fiber in the BLA to terminally inhibit the NB-BLA projection at the encoding stage in mice (Fig. S6A, B). The DIO-NpHR3.0 group exhibited reduced freezing time under the 10 kHz tone retrieval but the freezing time under the 1 kHz tone retrieval remained unchanged (Fig. S6C), thus leading to a reduced discrimination index compared to the control group. These results suggest that inhibition of NB-BLA projection only reduced the AFM but not the DAFM.

To conclude, we propose that, at the encoding stage, the ACh signal projects from the NB to the ACx via nAChR. The ACh signal then influences the reactivation of BLA ensembles by projecting from the tone-responsive ACx ensembles to BLA ensembles at the retrieval stage to control the DAFM. Our data demonstrate the crucial role of the tripartite NB^ACh^-ACx-BLA neural circuit in modulating DAFM.

## Conclusion and discussion

For animals to survive, they must react defensively in the presence of stimuli that resemble threats, not harmless stimuli. Being unable to discriminate AFM is abnormal and is related to certain psychiatric disorders [21]. In this study, we found that the cholinergic signal in the NB is required for the ACx clusters to discriminate shock-paired auditory signals from pure auditory signals. Particularly, the nicotinic ACh signal plays a crucial role in the modulation of DAFM, as nAChR blockers can diminish the increased magnitude of the ACx tone-responsive neuronal activity and reduce the DAFM. Furthermore, nAChR agonists can restore the reduced discriminative ability of AFM caused by inhibition of the NB cholinergic projections to the ACx. By using a neural activity-dependent tagging system to label engram cells, we demonstrated that the optogenetic inhibition of cholinergic projections of NB-ACx at the encoding stage obfuscates distinct tone-responsive neurons of ACx in recognizing fear-paired tone and fear-unpaired tone signals, while simultaneously increasing neuronal activity in fear-unpaired tone BLA engram cells at the retrieval stage. The results suggest that the tripartite neural circuit of NB^ACh^-ACx-BLA plays a crucial role in the modulation of DAFM.

Basal forebrain cholinergic neurons play a crucial role during fear memory’s encoding, retrieval, and extinction stages [6, 14, 22–24]. Cholinergic projections of NB-ACx may also play an important role in DAFM. It has been shown that ACx-projecting cholinergic neurons in the BF also modulate auditory cortical tuning and neural plasticity to support auditory fear learning [6, 14]. However, it is unclear how cholinergic signals in ACx projecting from NB are involved in AFM discrimination. Our work revealed that not only is the ACh signal in the ACx significantly increased at the encoding stage, but the ACh signal in the ACx also responds differently to discriminate fear-paired from fear-unpaired auditory signals during the retrieval stage of DAFM. Therefore, we believe that the ACh signal in the ACx distinguishes fear-paired signals from non-fear-paired signals. Optogenetic inhibition of cholinergic projections of the NB-ACx causes reduced DAFM in mice by causing an inability to distinguish fear-paired signals from fear-unpaired signals. However, photogenetic activation of cholinergic projections of NB-ACx did not improve fear discrimination in mice. This might be due to the aversive feature of fear memory. Because the shock-paired tone is a strong stimulus causing a strong fear response, optogenetic activation of ACx engram cells under the shock-coupled tone condition did not further increase the fear response. It could not further improve the discriminative ability of AFM in mice.

Acetylcholine receptors (AChRs), including nAChRs and mAChRs, play a role in the development and synaptic plasticity and participate in glutamatergic transmissions in many brain regions related to learning and memory [25, 26]. Previous work on fear conditioning and retention of extinction learning has shown that treatment of both mAChR and nAChR antagonists significantly reduces the retrieval of fear memory and significantly enhances the retention of extinction learning compared to the control group, which is unaffected by the treatment of mAChR antagonist alone [5]. Our results show that the nAChR, not the mAChR, plays a crucial role in modulating DAFM. Blocking nAChRs can reduce the magnitude of ACx tone-responsive neural activity after receiving fear memory, which causes the mice to be unable to distinguish fear-paired signals from fear-unpaired signals during the retrieval stage. Treatment of nAChR antagonist MCM also resulted in reduced DAFM. However, treatment of nAChR agonist nicotine can restore the reduced discriminative ability of AFM caused by inhibition of the NB cholinergic projection to the ACx. Jiang L et al. found that type III Neuregulin 1, a key regulator of presynaptic targeting of α7 nAChRs, is required in multiple aspects for the modulation of excitatory plasticity at cortical-BLA synapses [27]. They further revealed that cholinergic signaling could enhance the firing of putative BLA principal neurons and induce glutamatergic synaptic transmission in the BLA, thus controlling fear-conditioning behaviors [5]. A recent study also reported that α7-nAChR of astrocytes in the ACx could be activated by aversive sensory stimulation and be required for fear memory persistence [18]. Thus, whether ACh signaling would affect the synaptic plasticity of ACx tone-responsive neurons, and whether this effect would be realized through cholinergic receptors on neurons or through astrocytes to participate in DAFM, should be further studied.

Consistent with previous research [17], we also testified that different neuronal populations in the ACx correspond to different tone stimuli. Optogenetic manipulation of neuronal clusters that correspond to fear-unpaired signals does not affect fear memory. Though the reactivation of engram cells is a well-known phenomenon in the retrieval of fear memories [19, 20, 28], we found that BLA engram cells express low levels of reactivation in the presence of a fear-unpaired signal, which was modulated by tone-responsive neurons in the ACx. In addition, activation of distinct tone-responsive neuronal clusters in the ACx is regulated by the cholinergic signal from the NB. Optogenetic inhibition of cholinergic projections of the NB-ACx obfuscates distinct tone-responsive neurons of ACx in recognizing fear-paired tone and fear-unpaired tone signals, while simultaneously increasing neuronal activity and reactivation of BLA engram cells in fear-unpaired tone during the retrieval stage.

In conclusion, our work demonstrates that the NB cholinergic signal is required to discriminate fearful auditory information from fear-free auditory information during the encoding stage. The NB cholinergic signal also controls the BLA engram cells through the ACx to modulate DAFM. We show that the tripartite NB^ACh^-ACx-BLA neural circuit plays an important role in modulating DAFM. We suggest that the manipulation of nicotinic cholinergic signaling in specific terminal fields could shed light on novel treatments for deficiencies in discriminative fear memory associated with certain neurobiological diseases.

## Methods

### Animals

The Institutional Animal Care and Use Committee of the Beijing Institute of Technology in Beijing, China approved all surgical and experimental procedures. The following animals were used in this study: adult (2–4 months old) male wild-type (WT) mice (C57/BL6) and ChAT-cre mice (Jackson laboratory Stock no. 006140). Mice were group-housed in a 12/12 h light/ dark cycle (2–5 animals per cage) at a consistent ambient temperature of 23 ± 1 °C and humidity of 50 ± 5%. All experiments were performed during the light cycle. Food and water were accessed ad libitum. Littermates were randomly assigned to each condition by the experimenter.

### Viruses

AAVs particles (AAV9-c-fos-tTA, titer, 2.5 × 10^12^gc/ml; AAV9-TRE-Gcamp6s, titer, 3.1 × 10^12^gc/ml; AAV9-c-fos-rtTA, titer, 2.2 × 10^12^gc/ml; AAVretro-c-fos-rtTA, titer, 2.0 × 10^12^gc/ml;AAV9-Syn-Ach3.0, titer, 3.5 × 10^12^gc/ml; AAV9-TRE-EGFP, titer, 2.0 × 10^12^gc/ml; AAV9-TRE-NpHR3.0-EGFP, titer, 3.2 × 10^12^gc/ml; AAV9-TRE-oChIEF-mcherry, titer, 2.3 × 10^12^gc/ml; AAV9-TRE- chrimsonR-mcherry, titer, 3.8 × 10^12^gc/ml; AAV9-DIO-NpHR3.0-mcherry, titer, 3.0 × 10^12^gc/ml; AAV9-DIO-mcherry, titer, 2.5 × 10^12^gc/ml; AAV9-RAM-cre-ERT, titer, 2.7 × 10^12^gc/ml; AAV9- DIO-chrimsonR-mcherry, titer, 3.1 × 10^12^gc/ml;) were purchased from Taitool, Shanghai, China.

### Surgery

The animals were deeply anesthetized and placed in a stereotactic frame (RWD, Shenzhen, China). Ophthalmic ointment was applied to prevent dehydration. The virus injection procedure was described previously [29]. The optic fiber transmissivity was examined by measuring the light intensity as the laser launch, tip of the coupled fiber (diameter, 200 μm; Ferrule O.D, 1.25 mm; N. A., 0.37; length, 2.0 or 4.0 mm; OriginOpto Inc., China), and the fiber ferrule implant by optical power meter (PM121D, Thorlab, USA). Fiber ferules were selected based on optical transmissivity (>80%). Viruses were injected into the ACx region (AP: −2.6 mm; ML: ± 4.5 mm; DV: −1.0 mm), NB region (AP: −0.5 mm; ML: ± 1.7 mm; DV: −4 mm), and BLA region (AP: −1.7 mm; ML: ± 3.25 mm; DV: −3.8 mm), respectively. Samples with deviation in injection sites were excluded.

We measured the intensity of coupled fibers before they were connected to the implanted fiber ferrule and each animal daily. The intensity was adjusted to a certain power. In addition, the fiber ferrule was placed 50–100 μm above the viral injection site. The optical fiber was secured to the skull using jeweler′s screws and dental cement.

For fiber photometry recordings in the ACx, the mice were injected with 300 nl viruses of AAV9-Syn-ACh3.0 (Fig. 1A) in the ACx region (AP: −2.6 mm; ML: ± 4.5 mm; DV: −1.0 mm). An optical fiber was implanted in the ACx. Mice were administrated in behavioral tests 4 weeks after surgery.

For Pharmacology, a subset of mice used in behavioral tests underwent implantation of bilateral cannulas for drug delivery targeted to the ACx (Fig. 1E). Viruses were expressed for one week before behavioral test.

For fiber photometry recoding or immune-histochemical staining c-fos in the ACx or BLA region and optogenetic stimulation in the NB (AP: −0.5 mm; ML: ± 1.7 mm; DV: −4 mm) (Fig. 5) or the ACx (Figs. 2A, 4) concurrently, the mice were simultaneously injected with 300 nl viruses of AAV9-hsyn-Ach3.0 or AAV9-c-fos-rtTA & AAV9-TRE-EGFP (mixed viruses) in the ACx or AAV9-c-fos-tTA & AAV9-TRE-Gcamp6s (mixed viruses) or AAV9-c-fos-tTA& AAV9-TRE-EGFP (mixed viruses) in the BLA, and injected with 300 nl virus of AAV9-DIO-NpHR3.0-mcherry, or AAV9-DIO-chrimsonR-mcherry in the NB. An optical fiber was implanted in the ACx or ACx and BLA. Mice were administrated in behavioral tests 6 weeks after surgery. Mice were simultaneously injected with 300 nl viruses of AAV9-c-fos-tTA & AAV9-TRE-Gcamp6s (mixed viruses) in the BLA, and 300 nl viruses of AAV9-RAM-cre-ERT AAV9-RAM-cre-ERT & AAV9-DIO-NpHR3.0-mcherry (mixed viruses) in the ACx. An optical fiber was implanted in the BLA and ACx at an angle of 15 degrees (AP: −2.6 mm; ML: ± 4.9 mm; DV: −1.0 mm). Mice were administrated in behavioral tests 4 weeks after surgery.

For optogenetic stimulation of different tone-responsive neurons in the ACx, the mice were injected with 300 nl viruses of AAV9-c-fos-rtTA &AAV9-TRE-NpHR3.0-EGFP (mixed viruses, Fig. 3). An optical fiber was implanted in the ACx. Mice were administrated in behavioral tests 4 weeks after surgery.

Samples with deviation in injection sites were excluded.

### Activity-dependent neuronal labeling

For labeling ACx and BLA neurons, the AAV9-c-fos-rtTA system, AAV9-c-fos-tTA system, and AAV9-RAM-cre-ERT system were used [30, 31].

For the AAV9-c-fos-rtTA system, 250 µl Dox (dissolved in saline at 5 mg/ml) was intraperitoneally injected into the mice 2 h before behavioral tests [32].

For the AAV9-c-fos-tTA system, the mice were continuously fed with a Dox diet (40 mg/kg) 7 days before virus injection surgery. The mice were taken off Dox 48 h to open the labeling window and put back on Dox immediately after fear conditioning [32–34].

For the AAV9-RAM-cre-ERT system, we intraperitoneally injected tamoxifen (150 mg/kg, 250–300 µl) into the mice 24 h before behavioral tests. Tamoxifen was dissolved in soybean oil (Aladdin) at 20 mg/mL for 12 h in the dark at room temperature (22–24 °C) [17].

### Discriminative auditory fear conditioning

All mice were handled for 1 h per day for two weeks prior to the behavioral tests. The behavioral session was conducted in a fear conditioning box (46001, UGO BASILE S.r.l, Italy). Freezing was defined as a complete absence of movement, except for respiration. Scoring of the freezing response duration was started after one second of sustained freezing behavior [34]. The freezing was measured using the Anymaze (version 6.0, Stoelting) software based on a threshold of change in video image pixels. The mice were delivered with 10 kHz and 1 kHz tones and the percentage of freezing time was averaged for the tone each day.

The changing index of freezing was calculated using the equation:

$$The\,changing\,index = \left( {T_{light\,on\,freezing} - T_{light\,off\,freezing}} \right)/\left( {T_{light\,on\,freezing} + T_{light\,off\,freezing}} \right).$$

The discrimination index of fear memory was calculated using the equation:

$$The\,discrimination\,index = \left( {T_{10kHz\,freezing} - T_{1kHz\,freezing}} \right)/\left( {T_{10kHz\,freezing} + T_{1kHz\,freezing}} \right).$$

At the encoding stage (day 1), after 5 min of habituation in context A, the mice received 5 pairings of auditory tone (70 dB SPL 10 kHz or 1 kHz pure tone, 20 s duration) with electric foot shocks (1 mA, 2 s duration, and overlapped with the last 2 s of tone). The time interval between each trial was 70 s. In Fig. 1E, F, and S1C, 10 min before the test, 0.5 µl of pharmacological agent was administered simultaneously bilaterally at a rate of 0.125 µl every 10 s by cannula. The mice were administered either saline, mecamylamine (MCM, 10 µM, 20 µM Aladdin, 826-39-1), or atropine alone (10 µM, 20 µM Aladdin, 5908-99-6). In Fig. 2, each tone is accompanied by optogenetic inhibition or activation. In Fig. 2G, 30 min before the test, mice were intraperitoneally injected with saline and nicotine (10 µM, Sigma Aldrich), and each tone is accompanied by optogenetic inhibition.

At test 0 (day 2), to label tone-responsive ACx neurons, the mice were delivered three times of 10 kHz tone or three times 1 kHz tone.

At test 1a or test 1b (day 2 or day 3), the mice were tested for freezing behavior in response to tests 1a (new fear-unpaired 1 kHz or 10 kHz tone) and 1b (fear-paired 10 kHz or 1 kHz) with a time interval of 2 h in different contexts B and C. The mice were delivered three (Figs. 1, 2, 4, 5 and Figs. S1, 2G, H, 4) or four (Fig. 3, S2E, F) representations of both 10 kHz and 1 kHz tones. In groups with four representations of tones, the first and third tones were accompanied by optogenetic activation or inhibition. In Fig. S2A–C, the mice were only given 10 times optogenetic activations. For test 1, each group of mice was randomly assigned and then equally received 1 kHz or 10 kHz tone in different contexts B and C, and after 90 min, the mice were carefully perfused.

All contexts were different in shape, background, and floor texture.

### In vivo optogenetic stimulation

For yellow light inhibition, the mice were stimulated with 590 nm light (10 mW, constant light on).

For yellow light activation, the mice were stimulated with 590 nm light (1 mW, 20 Hz, 5 ms duration).

For blue light activation, the mice were stimulated with 470 nm light (1 mW, 20 Hz, 5 ms duration).

For all experiments, the light was performed 1 s before the sound was delivered and lasted 21 s [35].

### In vivo pharmacological stimulation

For intracranial cannula administration in bilateral ACx, mice were injected with drugs 10 min before the behavioral tests. 0.5 µl of the pharmacological agent was administered simultaneously bilaterally at a rate of 0.125 µl every 10 s.

For intraperitoneal administration, mice were injected with 100 µl of drugs 30 min before the behavioral tests.

### Immunofluorescence and analysis

The mice were treated as previously described [29]. In particular, the primary antibodies used were: anti-chat (1:1000, goat, AB144P, Millipore, USA), and anti-c-fos (1:1000, rabbit, 2250 s, Cell Signaling Technology, Inc.). Confocal fluorescence images were acquired using a Nikon A1 confocal laser scanning microscope with ×10, ×20 objectives for imaging stained or autofluorescent neurons. Images were analyzed by NIS-Elements AR and the labeled cells were counted by an experimenter blind to condition who analyzed the images using the automated ImageJ cell counter.

### Fiber photometry recording

The fiber photometry recording was performed in an apparatus obtained from the Thinker Tech Nanjing Biotech Limited Co., Nanjing, China. The signal was digitized and collected by the ThorCam-DAQ software. The calcium signal was recorded after 3 weeks of GCamP6s virus expression. The timing of behavioral variables was recorded by the same system.

To calculate the GCamP6s signal, the relative fluorescence changes of ΔF/F were calculated as Ca^2+^ signal, and the fluorescence responses of ACh signal were calculated as follows:

$$\Delta {{{\mathrm{F}}}}/{{{\mathrm{F}}}} = \left( {{{{\mathrm{F}}}}_{{{{\mathrm{raw}}}}} - {{{\mathrm{F}}}}_{{{{\mathrm{baseline}}}}}} \right)/{{{\mathrm{F}}}}_{{{{\mathrm{baseline}}}}}$$

### Statistical analysis and reproducibility

A two-way repeated measures ANOVA or Mixed-effects model was used to assess significant interactions for group comparisons. Bonferroni-corrected *P* values were used and indicated for multiple comparisons. For comparisons between two columns or two curves of fiber photometry, the unpaired two-tailed *t*-test was used. An ordinary one-way ANOVA was applied for comparisons among three or four columns.

All data were presented as means ± standard errors of the means (SEM). All statistical analyses were performed with GraphPad Prism 8.0. All statistical details of experiments can be found in the figure legends. Each experiment has been repeated three times in the manuscript with similar results.

## Supplementary information

supplementary figure legends

Figure s1

Figure s2

Figure s3

Figure s4

Figure s5

Figure s6

## Data Availability

All data needed to evaluate the conclusions are present in the manuscript and /or supplementary materials.
