# Supplementary material for: ACx-projecting cholinergic neurons in the NB influence the BLA ensembles to modulate the discrimination of auditory fear memory
Source: Transl Psychiatry. 2023 Mar 6;13:79. doi: 10.1038/s41398-023-02384-8 (PMC9988865; doi:10.1038/s41398-023-02384-8)
Supplement: Supplementary file 1 — supplementary figure legends [file 41398_2023_2384_MOESM1_ESM.docx]

**Fig S1. ACx is involved in the DAFM, related to Figure 1**

(**A)**The behavioral design. (**B**) The behavioral performance on day 1 (left) and day 2 (right) (n = 7). Higher percentage of freezing time occurred during the onset of the fear-related signal (1kHz). (**C and D**) Percentage of the freezing time (C) and the discrimination index (D) in mice under treat the nAChR blocker MCM in ACx at the encoding stage in 1kHz shock-paired experiment. (saline, n = 8; 10 µM, n = 8; 20 µM, n = 8; freezing: F (2, 30) = 11.56, P < 0.001; discrimination index: F (2, 21) = 17.32, P < 0.001). Statistical tests: **B** and **C,** freezing: two-way ANOVA with Bonferroni's multiple comparisons test. **D**, discrimination index: one-way ANOVA with Bonferroni's multiple comparisons test. ns, not significant; *P < 0.05, **P < 0.01, ***P < 0.001. Data are presented as mean ± SEM.

**Fig S2. NB cholinergic projection to the ACx is required for the DAFM, related to Figure 2.**

(**A**) Schematic of optogenetic stimulation and fluorescence recordings. In chat-cre mice, AAV9-DIO-chrimsonR-mcherry was infused into the NB and AAV9-Syn-Gcamp6s or AAV9-Syn-ACh3.0 were injected into the ACx. (**B and C**) Corresponding Ca^2+^ signal (B) and fluorescence response (C) of ACh3.0 in the ACx under activation of NB cholinergic neurons. (**D**) Percentage of the freezing time (left, middle, F (1, 20) = 9.731, P = 0.005; NpHR3.0, n = 6; mcherry, n = 6) and the discrimination index (right) in mice under inhibition of ACx-projecting cholinergic neurons of the NB at the encoding stage in 1kHz tone-paired foot shock experiment. (**E**) Percentage of the freezing time (left, middle) and discrimination index (right) in mice under either or both nicotine intraperitoneal injection and inhibition of ACx-projecting cholinergic neurons of NB at the encoding stage (mcherry-saline, n = 7; mcherry-nicotine, n = 8; NpHR3.0-saline, n = 8; NpHR3.0-nicotine, n = 8; freezing: F(3, 54) = 7.559, P < 0.001; discrimination index: F(3, 27) = 10.91, P < 0.001). (**F**) Schematic of virus injection of AAV9-DIO-NpHR3.0-mcherry in the NB of chat-cre mice. (**G**) Experimental strategy for optogenetic inhibition of ACx-projecting cholinergic neurons of the NB at retrieval stage (day 2). (**H**) Percentage of the freezing time (left, middle, F (1, 60) = 3.705, P = 0.06; NpHR3.0, n = 9; mcherry, n = 9) and the discrimination index (right).Statistical tests: **D**, **E** and **H** freezing: two-way ANOVA with Bonferroni's multiple comparisons test. **D** and **H**, discrimination index: unpaired two-tailed t-test. **E**, discrimination index: one-way ANOVA with Bonferroni's multiple comparisons test. ns, not significant; *P < 0.05, **P < 0.01, ***P < 0.001. Data are presented as mean ±SEM.

**Fig S3. The ACx ensembles are responsive to the DAFM, related to Figure 3**

(**A**) Representative confocal images of the ACx from mice injected with AAV9-c-fos-rtTA and AAV9-TRE-EGFP under different situations (blue, DAPI; green, the activated ACx cells at different situations were marked by EGFP). Scale bar: 200 μm. (**B**) The percentages of EGFP^+^ cells among DAPI^+^ cells. (**C**) Representative confocal images of the ACx from mice injected with AAV9-RAM-cre-ERT and AAV9-DIO-EGFP under different situations. Scale bar: 200 μm. (**D**) The percentages of EGFP^+^ cells among DAPI^+^ cells (blue, DAPI; green, the activated ACx cells at different situations were marked by EGFP). (**E**) Representative confocal images of the BLA ensembles from mice injected with AAV9-c-fos-tTA and AAV9-TRE-EGFP under the different situations. Scale bar: 200 μm. (**F**) The percentage of EGFP^+^ cells among DAPI^+^ cells (blue, DAPI; green, the activated BLA cells at different situations were marked by EGFP). (**G** and **H**) Percentage of the freezing time on day 1 (left) and day 2 (right) when inhibiting 10kHz-tone-responsive ACx neurons (**G**) or 1kHz-tone-responsive ACx neurons (**H**) following Fig 3B behavioral design. (**I** and **J**) Percentage of the freezing time on day 1 (left) and day 2 (right) when activating 10kHz-tone-responsive ACx neurons (**I**) or 1kHz-tone-responsive ACx neurons (**J**) following Fig 3B behavioral design. Statistical tests: **B**, **D** and **F**: one-way ANOVA with Bonferroni's multiple comparisons test. **G**, **H, I and J**: unpaired two-tailed t-test. ns, not significant; *P < 0.05, **P < 0.01, ***P < 0.001. Data are presented as mean ±SEM.

**Fig S4. Optogenetic manipulation of BLA-projecting tone-responsive neurons in the ACx regulates DAFM**

(**A**) Left: Schematic of virus injection. AAV9-c-fos-rtTA was injected into BLA and AAV9-TRE-NpHR3.0-mcherry or AAV9-TRE-chrimsonR-mcherry were infused into the ACx. (**B**) Behavioral design. (**C, D, E and F**) Schematic of optogenetic inhibition (left) and the percentage of the freezing time (right 1, 3) and the changing index (right 2, 4) in mice during 1kHz retrieval stage (right 1, 2) or 10kHz retrieval stage (right 3, 4) at day 3 when optogenetic inhibition of the 10kHz-tone responsive ACx neurons (**D**) and 1kHz-tone responsive ACx neurons (**F**). (**D**: NpHR3.0, n = 7, mcherry, n = 7, chrimsonR, n = 7; **F**: NpHR3.0, n = 7, mcherry, n = 7, chrimsonR, n= 7). Statistical tests: **D** and **F**, freezing: two-way ANOVA with Bonferroni's multiple comparisons test; changing index: one-way ANOVA with Bonferroni's multiple comparisons test. ns, not significant; *P < 0.05, **P < 0.01, ***P < 0.001. Data are presented as mean ±SEM.

**Figure S5**: **Cholinergic signaling influences the neural activity of tone-responsive neurons in ACx during the encoding stage**

(A) Left: schematic of virus injection of mixture virus of AAV9-c-fos-rtTA and AAV9-TRE-Gcamp6s in the ACx. (B) Behavioral design. (C, D) the mean and SEM of the fluorescence responses of Gcamp6s with MCM or saline injection in 10k Hz tone (C, MCM, n=6; saline, n=6) and 1k Hz tone (D, MCM, n=6; saline, n=5). Red line indicated bins between the pre-shock and post-shock trials with two-tailed unpaired t-test significance (P < 0.05). Data are presented as mean ±SEM.

**Fig S6. NB-BLA projection only affects the AFM**

(**A**) Schematic of virus injection. In chat-cre mice, AAV9-DIO-NpHR3.0-mcherry was infused into the NB and the optic fiber was implanted in the BLA. (**B**) Experimental design. (**C**) Percentage of the freezing time (left, middle) and the discrimination index (right) in mice under 1kHz-tone or 10kHz tone retrieval when inhibition of the specific NB-cholinergic neural terminal projecting to the BLA at the encoding stage (mcherry, n = 6; NpHR3.0, n = 6; freezing: F (1, 20) = 4.655, P = 0.04). Statistical tests: **C**, discrimination index: unpaired two-tailed t-test. **C**: freezing: two-way ANOVA with Bonferroni's multiple comparisons test. ns, not significant; *P < 0.05, **P < 0.01, ***P < 0.001. Data are presented as mean ±SEM.
